# The Geometry and Dynamics of Meaning

**DOI:** 10.1111/tops.12767

**Published:** 2024-11-10

**Authors:** Peter Gärdenfors

**Affiliations:** ^1^ Department of Philosophy and Cognitive Science Lund University

**Keywords:** Cognitive semantics, Word learning, Geometric representations, Conceptual spaces, Event cognition, Force dynamics

## Abstract

An enigma for human languages is that children learn to understand words in their mother tongue extremely fast. The cognitive sciences have not been able to fully understand the mechanisms behind this highly efficient learning process. In order to provide at least a partial answer to this problem, I have developed a cognitive model of the semantics of natural language in terms of conceptual spaces. I present a background to conceptual spaces and provide a brief summary of their main features, in particular how it handles learning of concepts. I then apply the model to give a geometric account of the semantics of different word classes. In particular, I propose a “single‐domain hypotheses” for the semantics of all word classes except nouns. These hypotheses provide a partial answer to the enigma of how words are learned. Next, a dynamic cognitive model of events is introduced that replaces and extends the function of thematic roles. I apply it to analyze the meanings of different kinds of verbs. I argue that the model also explains some aspects of syntactic structure. In particular, I propose that a sentence typically refers to an event. Some further applications of conceptual spaces are briefly presented.

## Abstract 2

This article presents my theory of conceptual space and uses it for modeling the semantics of natural language. A semantic analysis of word classes is presented together with a cognitive model of the structure of events.

## 1 An enigma

Knowing a language is not just knowing the words (lexicon) and the rules for putting them together (grammar) but primarily knowing the *meanings* of the words (semantics). We are not born with a language, so the mapping between words and their meanings must be learned. Children learn new words miraculously fast. A teenager understands 50,000–60,000 words of her mother tongue by the time she finishes high school. A simple calculation reveals that she has learned an average of 9–10 words *per day* during her childhood (Carey, 1978). No other form of learning is so efficient. Yet the underlying learning mechanisms are still to a large extent unknown.

Children's learning of words can be compared to that of artificial neural networks. Some systems, for example, ChatGPT, have become very good at communicating in ordinary language. These systems, however, must be trained on billions of examples before they function satisfactorily (and it is still questionable that they *understand* the texts). In contrast, a single example of how a word is used is often sufficient for a child to learn its meaning. The enigma is how the child's learning processes work.

A first cue is that words do not mean just anything. Our minds perceive different kinds of structures in the world, and word meanings, to a large extent, follow these structures. Another cue is that the syntactic markers of a new word often reveal its word class (Bloom, 2002). The markers help the child to look for an object when a noun is used, a property of an object when an adjective is used, an aspect of an event when a verb is used, and so forth.

In my research, I have been concerned with the semantics of natural language. During my logic days, I studied model‐theoretic semantics, including Montague semantics, but I found that there are many types of meanings of expressions in natural languages that are difficult to analyze in this type of semantics. Therefore, I turned to cognitive semantics. I found the notion of image schemas—as developed by Langacker, Lakoff, Talmy, Herskovits, and others—appealing. I realized, however, that there were very few constraints on what could be counted as an image schema. I have therefore aimed for a more principled account.

The results have been presented in my books *Conceptual Spaces* (2000) and *Geometry of Meaning* (2014). A main contribution of the second book is a semantic analysis of different word classes. Linguists typically define word classes in terms of their syntactic properties: nouns have plurals, verbs have tenses, and so forth. When word classes are taught at an introductory level in school, however, semantic criteria are used—for example, nouns stand for things, and verbs describe actions—but these criteria are seldom presented in a systematic and rigorous manner. My aim was to show that word classes can be given a (syntax‐free) semantic analysis based on cognitive and communicative constraints. In Section 2, I introduce conceptual spaces as a geometric way of representing meaning. In Section 3 below, the analysis of some of the main word classes is summarized.

The cognitive semantics initiated by Langacker (1987) and Lakoff (1987) focused on ordinary space as a foundational meaning structure. However, at an early stage, Talmy (1988) pointed out that *force* also plays an important role. When I describe the semantics of verbs, I generalize his force dynamics to verbs in general. Verbs can be divided into manner verbs and result verbs. The meaning of result verbs can be described in terms of vectors in property spaces. Manner verbs, however, require the force domain. In other words, both geometry and dynamics are required to develop a satisfactory cognitive theory of semantics.

Words do not occur in isolation but are typically combined into sentences. In the logical tradition, the meanings of sentences are defined with the aid of truth conditions. However, this is not very illuminating given how our minds understand the meaning of words. As an alternative approach, I have, together with Massimo Warglien, proposed a cognitive model of *events* (Gärdenfors & Warglien, 2012). The basic structure contains four components: agent, force vector, patient, and result vector. These elements are given structures with the aid of conceptual spaces (Gärdenfors & Warglien, 2012). The basic semantic thesis states that sentences typically express events (or states as special cases). The event model and some of its applications to semantics will be presented in Section 4. Section 5 summarizes my semantic program applying it to some fundamental questions in linguistics, and Section 6 briefly presents some further applications of the theory of conceptual spaces.

## 2 Conceptual spaces

The theory of conceptual spaces (Gärdenfors, 2000, 2014) builds on two central ideas about the structure of concepts: (i) information is sorted into *domains* such as space, force, color, and shape, and (ii) domains have a geometric or topological structure. The theory is a kind of neo‐Kantian project that aims at unveiling the fundamental structures of human thinking. For example, the domains of conceptual spaces can be seen as an extension of the “Anschauungsformen” introduced by Kant. Conceptual spaces are proposed as theoretical entities that are hypothesized to mirror cognitive structures.

### 2.1 Domains

In cognitive linguistics in general, the notion of a domain is central. Following Langacker (1987), there has been a strong tendency in the literature to interpret the notion of domain in an all‐encompassing way. Langacker (2008, p. 44) writes that “the term is broadly interpreted as indicating any kind of conception or realm of experience.” Together with Simone Löhndorf, I have argued that Langacker's concept of domain conflates several components invoked in the analysis of lexical meanings (Gärdenfors & Löhndorf, 2013). In particular, we find his distinction between locational and configurational domains misleading. His configurational domains are better seen as *meronomic* information concerning the relations between parts and wholes rather than something pertaining to domains.

I confine myself to domains that Langacker calls *dimensional*. This seems to be how “domain” is used in cognitive psychology. Even if we do not know much about the geometrical structures of many of the domains, it is quite obvious that there are several examples of nontrivial structures. Psychology has a long tradition of analyzing the dimensionality of perceptual structures. Shepard (1987) argues that, as soon as perception relating to a particular domain can be graded by *similarity*, mathematical techniques can be used to extract a low‐dimensional space representing similarity judgments. A large number of perceptual spaces have been identified in this manner.^1^

The domains are composed of *quality dimensions*. One‐dimensional domains include *height*, *temperature*, *time*, and *pitch*. However, many domains are multidimensional, for example, (physical) *space*, *color*, *taste*, and *force*.

**Fig. 1:**
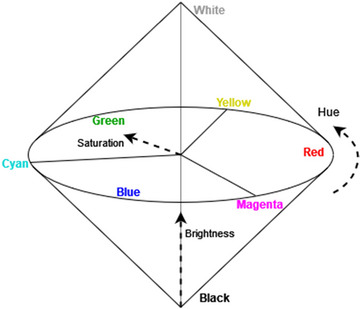
Color space.

The notion of a domain is defined as a set of integral dimensions that are separable from all other dimensions.^2^ For instance, color properties comprise three fundamental dimensions of color perception: hue, saturation, and brightness (see Fig. 1). A central thesis in the theory of conceptual spaces is that natural properties (such as colors) correspond to *convex* regions of a single domain. This is called “Criterion P” in Gärdenfors (2000). A region is convex when, for every pair of points *x* and *y* in the region, all points *between* them are also in the region. This thesis has received empirical support, for example, for color categories (Jäger, 2010).

The central notion of a conceptual space is defined as a collection of one or more domains together with a *distance function* that represents the similarity relations between objects: The closer two points are in the space, the more similar are the objects they represent.

Within this framework, objects are seen as instances of concepts and are mapped into points of the space, and concepts are represented as regions of the space.

### 2.2 Neuroscientific correlates

The cortex abounds in topographic maps, whereby neighborhood relations at the sensory periphery are preserved in the arrangement of neurons in various regions of the central nervous system. For example, one finds somatotopic maps representing sensory positions on the body; and there are tonotopic maps that transform the pitch dimension of a sound to a *spatial* dimension along the cochlea. The topographic maps correspond to distinct *domains* of representation since most of them preserve the modularity of the senses.

More recently, the hippocampal system has been proposed as a system for representing not only locations in ordinary space but for representing different kinds of conceptual spaces (Bellmund, Gärdenfors, Moser, & Doeller, 2018; Constantinescu, O'Reilly, & Behrens, 2016; Theves, Fernandez, & Doeller, 2019). The dynamic mapping system enables rapid reorganization of “coordinate systems” through remapping between orthogonal representations across different conceptual domains. The result is a multitude of cognitive spaces at different resolutions and hierarchical levels.

During navigation in ordinary space, place cells in the hippocampus show increased firing at a particular location within the space. Then grid cells in the adjacent entorhinal cortex fire at multiple locations within an environment, and these locations form a hexagonal grid. Combining the information from place cells and grid cells, these cells generate self‐localization. The coding also supports geometric computations when planning spatial navigation by representations of distances and directions.

Several studies have shown that similar mechanisms are exploited in tasks involving other conceptual spaces (Zheng et al., 2024). An example is presented in Nitsch et al. (2024) who study how a two‐dimensional value space used in a decision task becomes represented in the brain. The result is that the entorhinal cortex exhibits a grid‐like representation with an orientation aligned to the axis through the value space that is most relevant for choices. These experiments suggest that the grid cells function as a “universal coordinate system” that can be used to represent a number of different conceptual spaces.

### 2.3 Properties and concepts

I make a technical distinction between *properties*, which are convex regions of a single domain, and *concepts*, which are complexes of convex regions in several domains. As I will argue in the following section, adjectives typically refer to single domains, whereas the meaning of most nouns in natural language can only be described as clusters of properties in several domains.

As a paradigm example of an object category, consider “dog.” When we encounter dogs as children, the main domains that we learn about are shape, size, sound, smell, color, and texture. We also learn about the *material* of different categories, for example, that dogs like other animals consist of skin, flesh, and bones. In parallel, we learn about other domains of dogs such as their typical behavior (the action domain), biological characteristics, and their roles in human society. We also learn about the parts of a dog and how they are related.

Even if several domains are involved in representing a category, not all domains have the same *salience* when determining object similarity. For example, the shape of a dog is more salient than its smell. The salience of a domain can be defined as the degree of attention that is given to the domain. This means that salience is context‐dependent. To model salience, I assume that the representation of a category also contains information about the *weights* of the domains (see Gärdenfors, 2000, Section 4.2). These weights are used to determine the distance function in the space and the degree of similarity between different objects. Because a property is assigned a particular domain, the salience of the domain can then be used as a measure of how characteristic a property for a particular category is. This construction implies an intimate connection between similarity judgments and characteristic properties (Gärdenfors & Osta‐Vélez, 2023).

### 2.4 Connections to prototype theory

The criterion that properties and concepts are represented by convex regions derives independent support from the *prototype theory* of categorization developed by Rosch and her collaborators (e.g., see Hampton, 2007; Lakoff, 1987; Mervis & Rosch, 1981; Rosch, 1975). The main idea of prototype theory is that within a category of objects, like those instantiating a property or a concept, certain members are judged to be more representative of the category than others. For example, robins are judged to be more representative of the category “bird” than are ravens, penguins, and emus. The most representative members of a category are called prototypical members.

When natural properties are defined as convex regions of a conceptual space, prototype effects are indeed to be expected. In a convex region, one can describe positions as being more or less central. In particular, if the space has a metric, one can calculate the center of gravity of a region. By using the metric of a conceptual space, one can thereby represent properties in the space as more or less *typical* of the categories according to their distance to the prototype (Hampton, 2007; Osta‐Vélez & Gärdenfors, 2022a).

**Fig. 2:**
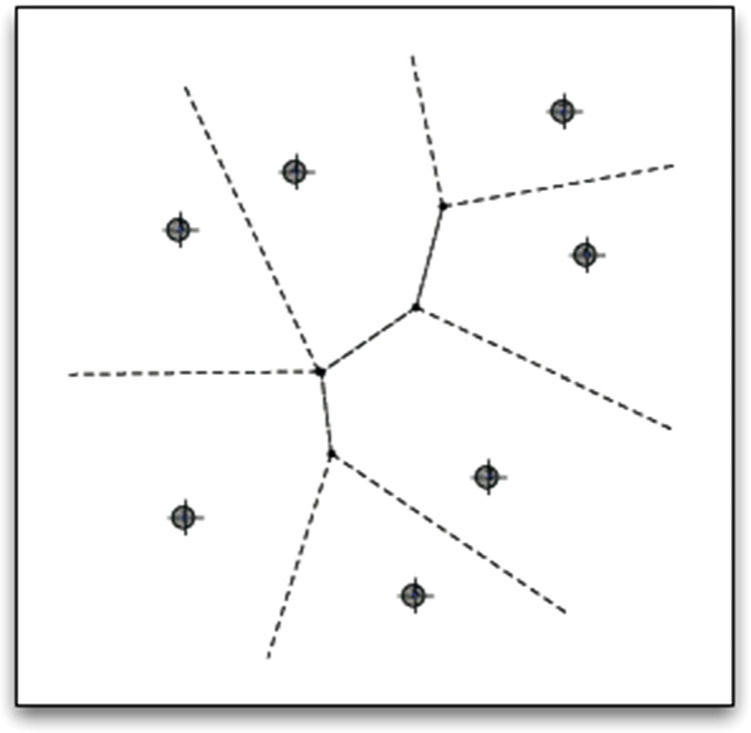
Voronoi tessellation of the plane into convex sets.

It is possible to argue in the converse direction too and show that if prototype theory is adopted, then the representation of properties as convex regions is to be expected, at least in metric spaces. Assume that some quality dimensions of a conceptual space *S* are given, for example, the dimensions of color space, and that one wants to partition it into a number of categories, for example, color categories. If one starts from a set of prototypes *p*_1_, …, *p*_*n*_ of the categories, for example, the focal colors, then these should be the central points in the categories they represent. If it is assumed that *S* is a metric space, the information about prototypes can be used to generate a categorization. In order to see this, assume that *S* is equipped with the Euclidean metric so that for every point *p* in the space, one can measure the distance from *p* to each of the prototypes. If one now stipulates that *p* belongs to the same category as the *closest* prototype, it can be shown that this rule will generate a partitioning of the space—the so called *Voronoi tessellation*. An illustration of the Voronoi tessellation is given in Fig. 2.

A crucial property of the Voronoi partitioning of a conceptual space is that the Voronoi tessellation always results in a partitioning of the space into *convex* regions (see Okabe, Boots, & Sugihara, 1992).

### 2.5 A model of how concepts are learned

Prototype theory does not explain how prototype effects arise as a result of learning to use concepts. The theory can neither account for how new concepts can be created from relevant exemplars nor explain how the extensions of concepts are changed as new concepts in the same category are learned. I next outline how conceptual spaces can, at least partially, explain the enigma of word learning. The partitioning of the domains of a conceptual space is not innate, so the relevant regions of a conceptual space must be created with the aid of the experience of a language learner. In order to be useful, the concepts must not only be applicable to known cases but should *generalize* to new situations as well.

Learning a concept often proceeds by generalizing from a limited number of exemplars of the concept (e.g., see Langley, 1996; Nosofsky, 1988; Reed, 1972). Adopting the idea that concepts have prototypes, we can assume that a typical instance of the concept is extracted from these exemplars. If the exemplars are described as points in a conceptual space, a simple rule that can be employed for calculating the prototype from a class of exemplars is that the point p representing the prototype is defined to be the *mean* of all the exemplars (Langley, 1996, p. 99). The prototypes defined in this way can then be used to generate a Voronoi tessellation.

**Fig. 3:**
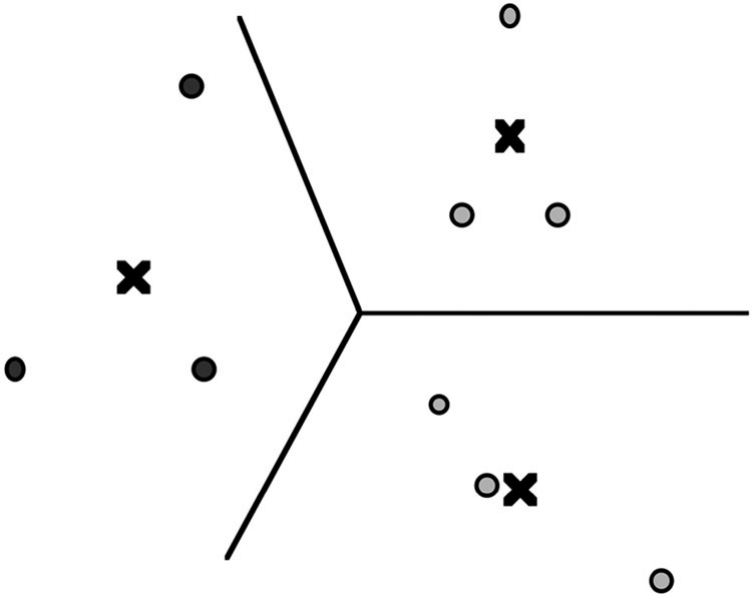
Voronoi tessellation generated by three classes of exemplars.

Applying this rule means that a prototype is not assumed to be given a priori but is determined by the experience of the subject. Fig. 3 shows a set of nine examples (represented as differently filled circles), grouped into three categories. Taking the means of the three groups generates three prototypical points (represented as black crosses) in the space. These prototypes then determine a Voronoi tessellation of the space.

The learning mechanism illustrated here shows how the application of concepts can be generalized on the basis of only a few examples of each concept. The additional information that is required for the generalization is extracted from the geometric structure of the underlying conceptual space that is required for the calculation of prototypes and for the Voronoi tessellation. In this way, the geometrical structure of conceptual spaces adds information to what is given by experience.^3^ The learning mechanism outlined here provides a cue to how the meaning of words can be learned so quickly.

## 3 Geometry of word meanings

Why are there words at all? Why do we not communicate with wordless songs like birds and whales do? The short answer is that we have words because we need to communicate about *concepts*—above all object, action, and space concepts (Carey, 2009; Spelke, 2000).

My primary application of conceptual spaces has been as a tool for representing the meanings of words in natural languages. In this section, I summarize the analysis of some of the main word classes in Gärdenfors (2014). Different languages sort up words in different ways. For lack of knowledge of other language groups, I here study the main word classes of Indo‐European languages. I focus on basic words that children learn early in their development, leaving the analysis of more abstract words for later studies.

### 3.1 Adjectives

The key idea here is that basic adjectives express properties, such as color, shapes, and sizes. Given my definition of properties, this motivates the following thesis:

*Single‐domain thesis for adjectives*: The meaning of an adjective can be represented as a convex region within a single domain.

For example, I hypothesized in Gärdenfors (2000) that all color terms in natural languages express convex regions with respect to the color domain. This means, for example, that there should be no language that has a single word for the colors denoted by “green or orange” since such a word would represent two disjoint areas in the color domain. Strong support for this conjecture was obtained by Jäger (2010; see also Douven & Gärdenfors, 2020; Regier, Kemp, & Kay, 2015). Furthermore, the single‐domain thesis implies that there is, for example, no adjective that means “long and hot” since such a word would involve two different domains.

Adjectives are also used for comparing things: Most languages have comparatives such as *taller* and *smarter* that apply to one‐dimensional domains. Many languages also have superlatives, for example, “tallest” and “smartest,” which can be defined from comparatives.

### 3.2 Nouns

Categories of ordinary objects can be seen as concepts that involve several domains, in contrast to properties. For example, an apple has a size, a shape, a color, a taste, a weight, and so forth. I consider such categories as the most basic meanings of nouns. The first nouns that children learn are of this type.

To be sure, there are nouns referring to many other types of entities. For example, abstract nouns have no physical referents, such as *law*, *retirement*, *inflation*, and *mind*. Lyons (1977, pp. 442–445), distinguishes three fundamental “orders”: (1) physical objects; (2) events, processes, states of affairs; and (3) propositions, schemas, and so forth that “are outside space and time.”^4^ The nouns in (2) and (3) require meaning domains other than those used for the object categories in (1).

More formally, a noun would be represented as a product of convex regions in several domains. Information about the *salience* of different domains is also included in the representation of the meaning of a noun. Langacker (1987) calls this “profiling” of the domain. For example, “roe” and “caviar” both denote fish eggs, but “roe” makes the biological aspect more salient and “caviar” brings forward the food domain. More trivial examples are that fruits do not include the emotion domain, and abstract nouns do not include the material domain, so these domains are not included in the representation of the concepts.

For most nouns, the domains that compose them can be correlated in different ways. For example, in the case of “fruit,” properties in the domains of *size* and *weight*, or *ripeness, color*, and *taste* co‐vary. Osta‐Vélez and Gärdenfors (n.d.) propose that the more co‐variation one finds between domains associated with an object concept, the more *coherent* the concept is judged to be. In general, natural kinds are judged to be more coherent than artifacts (Gelman, 2003). If a concept, say “bird,” has clusters of correlated properties, for example, “flies,” “feathers,” “wings,” and “beak” for the concept “bird,” then the coherence value will be high. Such clusters are typically not found for artifacts.

Even among nouns denoting physical objects, one can find different types. An important semantic distinction is that between *mass nouns* such as “silver,” “sand,” and “water” and *count nouns* that refer to countable objects such as “car,” “house,” and “flower.” Some nouns can be used in both ways: Compare “There are five apples in the bowl” (count use) with “Put two cups of apple on top of the pie pastry” (mass use). My account for the mass–count distinction is that for mass nouns, the *material domain* becomes the most salient—the shape domain is of minor importance. For example, “apple” as a mass noun just concerns the material. For count nouns, the opposite holds. This is another example of how varying the salience of domains can change the meaning of a word.

For abstract nouns, neither the material domain nor the location in space is relevant. For nouns denoting places, only the space domain is relevant. These examples indicate that nouns can be given a semantic classification by considering which domains are salient and which are irrelevant.

The analysis of the domains that are salient for different nouns can also explain why certain combinations of adjectives and nouns are semantically awkward. For example, “round silver” is difficult to interpret since the shape domain is irrelevant for mass noun “silver.” Similarly, “happy apple” is infelicitous since for “apple,” the emotion domain is irrelevant.

### 3.3 Verbs

When describing the semantics of verbs, it is also useful to consider the domains involved. Traditionally (Levin & Rappaport Hovav, 1991; Talmy, 1975, 1985), there have been two main ways of dividing verbs:

1. manner versus path as in *jog* versus *cross*; and
2. manner versus result as in *wipe* versus *clean*.

Levin and Rappaport Hovav (Levin & Rappaport Hovav, 2013; Rappaport Hovav & Levin, 2010; see also Talmy, 2001, Ch. 1) simplify the two divisions to just one by distinguishing between *manner verbs* and *result verbs—*where “manner verbs specify as part of their meaning a manner of carrying out an action, while result verbs specify the coming about of a result state” (Rappaport Hovav & Levin, 2010, p. 21).

In terms of domains, manner verbs denote *actions*. Following my earlier analysis of actions (Gärdenfors, 2007, 2014), the *force* domain is salient. For example, “push” can be described by the force vector exerted by an agent. In contrast, result verbs denote changes in location (path verbs), that is the space domain, or changes in object properties, which make domains for object categories salient. For example, “move” refers to changes in the spatial domain of the result vector, and “heat” refers to changes in the temperature domain.^5^ The changes can be expressed as vectors from the initial state in the domain to the end state.

The key thesis for the semantics of verbs can be summarized as follows:

*Single‐domain thesis for verbs*: Manner verbs refer to convex regions in force domain and results verbs to convex sets of vectors in the space domain or in some object category domain.

The single‐domain thesis for verbs is analogous to the single‐domain thesis for adjectives. Again, I submit that the regions of the spaces are convex. This constraint entails that there are no verbs that mean ‘walk and burn’ (multiple domains) and there are no verbs that mean ‘crawl or run’ (not convex).

The single‐domain thesis together with the classification of domains can explain why manner and result verbs fall out as natural classes. A consequence of the thesis is that the manner/result distinction is basically a cause/effect distinction: manner verbs refer to causes and result verbs to effects.

An important question is how the meaning of manner verbs can be expressed with the aid of conceptual spaces. One idea comes from Marr and Vaina (1982), who extend Marr and Nishihara's (1978) cylinder models to an analysis of actions. In Marr and Vaina's model, an action is described via differential equations for movements of the body parts of, say, a walking human. Applying Newtonian mechanics, it is clear that these equations can be derived from the *forces* that are applied to the legs, arms, and other moving parts of the body.

Even though our cognition may not be built precisely for Newtonian mechanics, I believe that our brains are made for extracting the forces that lie behind different kinds of movements and action (see Section 4.1). In accordance with this, I submit that the fundamental cognitive representation of an action consists of the pattern of forces that generates it (Gärdenfors, 2007; Gärdenfors & Warglien, 2012). For example, the force pattern involved in movements when somebody runs is different from the pattern of a person walking; and the force pattern for saluting is different from that of throwing (Gharaee, Gärdenfors, & Johnsson, 2017; Malt et al., 2014; Vaina & Bennour, 1985). However, it should be emphasized that the “forces” represented by the brain are theoretical constructs and not the scientific dimension introduced by Newton.

### 3.4 Prepositions

Most prepositions can be grouped into two classes: *locative*, indicating where something *is*, and *directional*, indicating where something *is going*. Locative prepositions specify the *location* (a region) in the spatial domain. Another function is fulfilled by directional prepositions. In a sentence such as “Oscar went *to* the library,” the phrase “to the library” has the same semantic function as a result verb: it specifies a *result vector* of an event (see Section 4.1). My proposal is that locative prepositions are represented by convex sets of points and directional prepositions by convex sets of paths.

Locational and directional prepositions depend on the spatial domain. In general, this domain is represented with the aid of the Cartesian coordinates representing width, depth, and height and where distances are measured using a Euclidean metric. However, there is another way of representing space, namely, in terms of *polar* coordinates that represent points in space in terms of distance from an origo and horizontal and vertical angles. A new definition of “between,” and thereby convexity, that differs from standard Euclidean betweenness can be defined in terms of polar coordinates (concerning the technical details, see Gärdenfors, 2014, Ch. 11; Zwarts & Gärdenfors, 2016). For example, the set of points of a semicircle around the origo form a convex set with respect to polar convexity. Given this definition of convexity, it can be shown that most locative prepositions, such as “inside,” “outside,” “near,” “far,” “in front of,” and “behind,” can be represented by a convex set of points.

Similarly, a betweenness relation for paths is easy to define (Zwarts & Gärdenfors, 2016) and thereby convexity of sets of paths. On the basis of such a definition, it is easy to show that the meaning of directional prepositions, for example, “to,” “from,” “into,” “out of,” “through,” “along,” and “across,” correspond to convex sets of paths.

In contrast to many analyses within linguistics (Herskovits, 1986; Zwarts & Winter, 2000), I argue that prepositions do not only refer to the spatial domain. Some prepositions refer to the *time* domain. In English, the most common ones are “before” and “after.” In addition, most typical uses of the prepositions “against,” “over,” “on,” and “in” depend on the *force* domain (for arguments, see Gärdenfors, 2014, Ch. 11; Vandeloise, 1991).

**Fig. 4:**
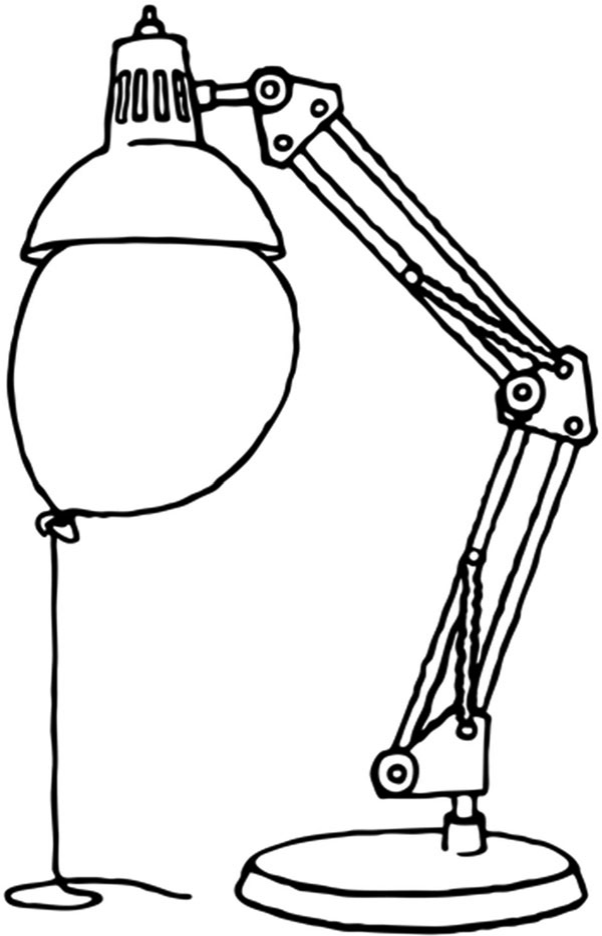
Is the lamp *on* the balloon?

For the preposition “on,” the semantic representation involves contact and support from below. A spatial region is not sufficient to determine the meaning of “on.” I propose that the meaning of “x is *on* y” is that the force vector from x makes x come in contact with y and a counterforce from y balances the force vector. Typically, this force vector is generated by gravitation. Thus, the force dimension is required for modeling the meaning of “on.” The interplay of the force and the counterforce is illustrated in Fig. 4, where one would hesitate to say that the lamp is on the balloon since the force relation is reversed when compared to the typical use of “on.”

In line with the analyses of the semantics of adjectives and verbs, I therefore put forward the following thesis:

*Single‐domain thesis for prepositions*: Prepositions represent convex sets of points, paths or vectors in a single domain.

Here, I have discussed the basic meanings of prepositions and assigned them single domains. Prepositions are, however, often used metaphorically and then the domains shift. For example, in “We meet at noon,” the basic spatial domain of ‘at’ is changed metaphorically to the time domain, and in “Oscar is in pain,” the domain of ‘in’ is changed to the emotion domain so that Oscar's experience is located in the region representing pain. The ubiquitous metaphorical uses of prepositions make them more difficult to analyze.

### 3.5 Adverbs

In the semantic model of verbs presented above, verbs refer to vectors. Vectors can vary in terms of dimension, orientation, and magnitude. Therefore, adverbs that are modifiers of verbs should refer to change in these features. For example, in “I sing slowly,” the adverb selects one of the several dimensions from the sound domain of *sing*. “I sing loudly” selects another. In “I jumped forwards” the adverb refers to the orientation of my motion. Finally, in “Victoria pushed the cart strongly,” the magnitude of the force vector representing push is strengthened by the adverb. When an action involves a pattern of forces, adverbs can modify the whole pattern by providing dynamic information, for example, “Victoria walked limply,” “Oscar smiled wryly,” or “Victoria kicked aggressively.” What is common to these examples is that the adverb *restricts* the regions associated with the meanings of the manner verbs. Similarly, in relation to a result verb describing a concatenation of changes (as in a path), an adverb can provide information about the form the path takes, for example, “Victoria crossed the football pitch crookedly.” In brief, the function of adverbs modifying verbs is parallel to how adjectives modify nouns.

As long as adverbs function as multipliers (diminishers or magnifiers) within a particular domain, the convexity principle can be defended. For example, if certain voice volumes *v*_1_ and *v*_2_ both count as speaking *loudly*, then any volume between *v*_1_ and *v*_2_ will also count as loudly. And for adverbs expressing the form of a path, path convexity as mentioned in Section 3.4 can be applied. So for adverbs modifying verbs, a single‐domain hypothesis can be defended. It is an open question whether the convexity principle can also be applied to other adverbs and whether a single‐domain hypothesis holds also for them. One limitation of the single‐domain thesis is that adverbs modifying adjectives can be zero‐dimensional, for example, “very” and “completely.” Such adverbs can therefore be associated with any domain.

### 3.6 Demonstratives

Languages vary a lot in terms of what demonstratives they contain (Diessel, 1999). Some languages have only location demonstratives (“here” and “there”), some also have object nearness demonstratives (“this” and “that”), but some, for example, Croatian, contain a very elaborate system of demonstratives involving several domains. Gärdenfors and Brala‐Vukanović (2018) argue that the meanings of demonstratives and articles can be analyzed in terms of a combination of the spatial domain—which is required for the deictic aspect—and a small set of other domains. They can be seen as a combination of an adjective with a deictic component. So also for this word class, a form of a single‐domain hypothesis can be put forward.

### 3.7 The role of the single‐domain hypotheses

To sum up this section, I have tried to argue for the following general semantic rule:

*Universal single‐domain thesis*: Words in all content word classes, except for nouns, refer to a single domain.

I am not certain how far I can push this thesis. To a large extent, its validity depends on how abstract domains are described. Nevertheless, I want to put forward the thesis as a strong heuristic that language learners (implicitly) apply when learning the meaning of a new word. A default rule is that if a newly encountered word is not a noun, then its meaning depends only on a single domain. This rule will simplify the identification of its meaning for the learner (cf., Bloom, 2002).

Another important aspect of the separate single‐domain theses is that they are *empirically testable*. Even if they turn out not to be valid for all words in a particular word class, they can still be valid for the first words that children learn in the class and thereby support the theses as learning heuristics.

## 4 The event model and its role in semantics

The geometrical aspects of semantics are apparent in the structure of the domains, with convexity as a general organizing principle for concept meanings. However, for the analysis of manner verbs and for some prepositions, the force domain is salient. For these areas, *dynamics* becomes a central semantic feature. Talmy (1988) is the locus classicus for the role of force dynamics but also Vandeloise (1991) who brought in forces in his analysis of prepositions.

In the previous section, I dealt with the semantics of separate word classes. This section turns to the semantics of sentences. In Gärdenfors (2014), I proposed that sentences refer to *events* (or states as a special case). In order to give support to this proposal, I must introduce a cognitive model of events that I have developed in collaboration with Massimo Warglien (Gärdenfors, 2014, 2024; Gärdenfors & Warglien, 2012).

### 4.1 The central elements

When describing events, linguists often resort to a thematic role structure where the basic roles are agent and patient (Dowty, 1991; Gisborne & Donaldson, 2019; Levin & Rappaport Hovav, 2005; Rissman & Majid, 2019). Some researchers view thematic roles as primitive semantic notions, but Gisborne and Donaldson (2019) give several arguments against this position (see also Langacker, 2008, on “conceptual archetypes”). In contrast, I argue that the event structure is primitive and that thematic roles can be derived from it (see also Gisborne & Davidson, 2019, p. 239). Interestingly, force and result vectors are almost never included among the roles.

**Fig. 5:**
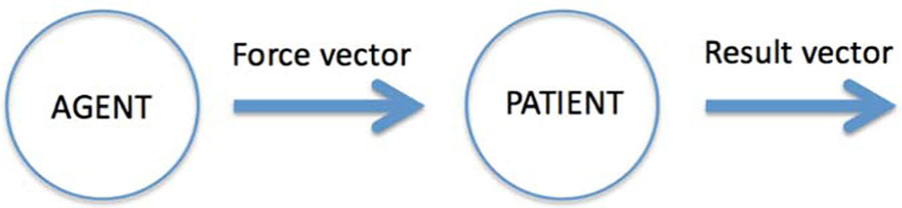
The four basic components of an event.

A prototypical event contains four basic components: an *agent*, a *patient*, a *force vector* and a *result vector*. These components are connected so that the action of an agent generates a force vector (more generally a force pattern) that affects a patient, causing a change in the state of the patient, which is described by the result vector. Fig. 5 illustrates the basic event schema.

**Fig. 6:**
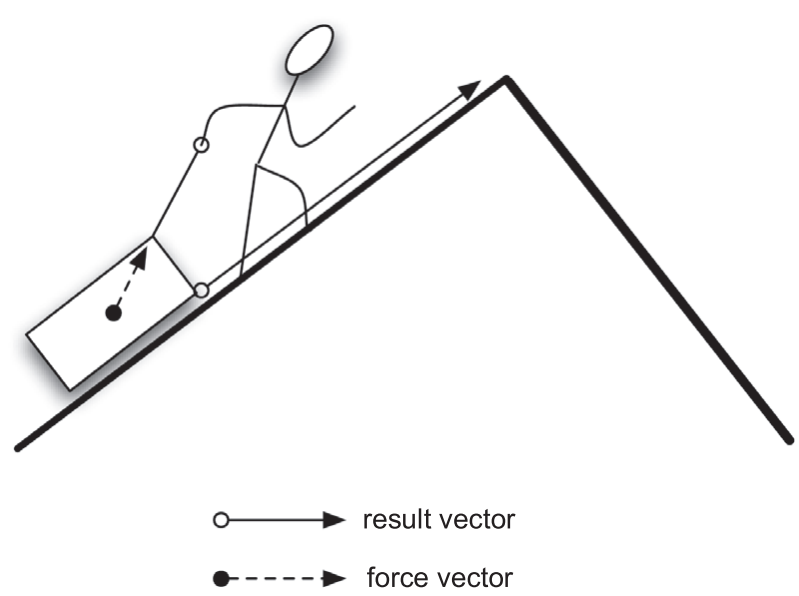
The vectors involved in the event of Oscar pulling a sledge to the top of the hill.

As a simple example, consider the event of Oscar pulling a sled to the top of the hill (see Fig. 6). In this example, the force vector of the pulling is generated by an agent (Oscar). The result vector is a change in the location of the patient—the sled (and, perhaps, a change in some other of its properties, e.g., it is getting wet). The result depends on the properties of the patient along with other aspects of the surrounding world: In the depicted event, for example, gravitation and friction act as *counterforces* to the force vector generated by Oscar. Another event is a ball rolling down a hill. The ball is the patient, but in this case, there is no agent. The force vector is generated by gravitation together with counterforces such as friction and support from the ground. The result vector is the movement of the ball.^6^

The event model that I present here is similar to the image schemas used within cognitive semantics. In particular, it relates to the force dynamics proposed by Talmy (1988), Wolff (2007, 2008), Croft (2012), and Copley (2019). The model aims at capturing (in a spirit similar to that of Dowty, 1991) the structure of a prototypical event. This means that events sometimes include additional features such as counterforces, instruments, and recipients. The event model is proposed as atheoretical entity that is hypothesized to mirror cognitive structures.

Following Gärdenfors (2014, 2020), the four main components of an event can be explicated in terms of conceptual spaces. In brief, this involves modeling agent and patient as object categories using various property domains, such as size, shape, color, weight, temperature, goal, and so forth. Each of these spaces has its own geometric or topological structure. Force vectors are represented in a three‐dimensional force space. Result vectors describe changes in properties and are thus vectors within a domain. More detailed descriptions of these components of conceptual spaces are presented in Gärdenfors (2020).

The structure of an event is determined by the mapping from the force vector to the result vector. The central object of an event is the patient. (In the linguistic literature, it is commonly called *theme*.) The force vector can more generally be described as a force pattern. Similarly, the result vector can be described as a sequence of changes, that is, as a *result path*. For example, if the event is Victoria walking to the library, her trajectory can be broken down into a sequence of smaller segments (depending on the desired level of granularity).^7^

When determining the result vector, not only the force vector F_A_ of the agent but also the *counterforces* must be taken into account. Often it is the patient that exerts a counterforce F_P_, for example by different forms of resistance. Such counterforces are at the center of Talmy's (1988) analysis of the role of force dynamics in verb semantics. A counterforce can, however, also be external to the agent and the patient as in the case of gravitation in the example above. The resultant force vector F_R_ that is the sum of F_A_ and all the counterforces is what determines the result vector. In Gärdenfors (2024), I analyze different combinations of agent force vectors and counterforces along the lines of Talmy (1988) and Wolff (2007, 2008).

Another component that is sometimes added to an event model is *instrument*. The general function of instruments is to modify the forces exerted by the agent. Using a hammer, for example, magnifies the forces of the agent's arm, using a spanner magnifies the turning forces of the agent, and using a loudspeaker magnifies the sound of the agent. This means that the force vector of the event is determined as a combination of the forces exerted by the agent and the modifications produced by the instrument.

It should be emphasized that the force and result vectors do not occur in a vacuum but are always construed in relation to a domain of a conceptual space, and thus inherit all the structural domain information. For example, since the domain of temperature is one‐dimensional, the heating vector has only one direction, while in the three‐dimensional domain of color, multiple directions of the result vector are possible. Grounding the vectors in conceptual spaces gives the event model a richer structure in comparison to previous models (e.g., that of Croft, 2012).

Furthermore, the mathematical properties of vectors contribute to an understanding of the structure of events. First, vectors can be more or less close to one another in the conceptual space they are defined for (Gärdenfors, 2000, 2014). For force vectors, this corresponds to the similarity of actions (via the closeness of the corresponding force vectors or force patterns). In a parallel way, the similarity of result vectors can be determined. Warglien, Gärdenfors, and Westera (2012) argue that similarities of vectors provide a natural explanation of similarities of verb meanings. As far as I know, no other theory of verb semantics can explain such similarities.

Second, vectors can be added and multiplied. The force vector acting on a patient can be added to a counterforce exerted by the patient. Furthermore, in many cases, a force vector is balanced by a counterforce resulting in a state instead of a dynamic event (e.g., when a door is prevented from being opened).

Third, sets of vectors can form convex sets. In particular, Gärdenfors and Warglien (2012) define an action category as a convex set of force vectors (force patterns). Since convexity has also been used to characterize properties and object categories (Gärdenfors, 2000, 2014), it is natural to propose that action concepts share a similar structure (Hemeren, 2008, p. 25). Indeed, there are strong reasons to believe that action concepts exhibit many of the prototype effects that Rosch (1975) presented for object concepts. In support of this, Hemeren (2008) and Malt et al. (2014) have shown that action concepts show a similar hierarchical structure and have similar typicality effects as object concepts.

### 4.2 Some connections to syntax

The structure of the event model has the potential to explain many general syntactic features. Here, I will only briefly outline some general aspects. First of all, when describing an event, agents and patients are typically expressed by nouns or noun phrases and actions and results by verbs or verb phrases. The event model thus explains the basic distinction between noun phrases and verb phrases. In contrast to the Chomskian syntactic approach in mainstream linguistics, this distinction is made on a semantic basis derived from the cognitive representation of events.

Second, the distinction between force vectors and result vectors explains the distinction between manner verbs and result verbs (Levin & Rappaport Hovav, 2005). Path verbs can be grouped together with verbs that describe property changes because of the tendency to give the same linguistic construction to a changing entity as to a moving one (Gruber, 1965; Jackendoff, 1972; Pinker, 1979): Both involve changes of properties of the patient, which manner verbs do not. Rappaport Hovav and Levin (2010) derive their thesis from their ACT‐BECOME model of events together with the constraint that a verb root can only be associated with one primitive predicate in an event schema as either an argument or a modifier. Since they assume that manner roots modify the predicate ACT and result roots are arguments of BECOME, the manner/result complementarity follows (Rappaport Hovav & Levin, 2010, Section 2). The distinction between force and result vectors corresponds quite clearly to their ACT and BECOME, but it adds a grounding in conceptual spaces that allows more predictions, for example, concerning the similarities of verb meanings.

Third, intransitive constructions such as “Victoria walks” and “Oscar jumps,” typically occur when the patient is *identical* to the agent. In this case, the agent exerts a force on itself. In other words, the agent modifies its own position in agent space (= patient space).

There exists no simple mapping between the role taken in an event and the designation of subject, object, or oblique in a sentence. In English (and many other languages), the most focused role is the designated subject, and the secondary focus is the designated object. In agreement with Givón (2001, p. 198), I view the selection of focus not as directly part of event representation but as a central element of the construal process. Consequently, the construals are contextual, depending on what the conversation partner already knows or believes or will find most interesting.

For example, consider an event where Victoria (agent) scrubs (action) a table (patient) clean (result). This event can result in a number of different construals (it is marked which type of verb is used):

“Victoria scrubs” (agent, manner, intransitive).

“Victoria cleans” (agent, result, intransitive).

“The table was cleaned” (patient, result, passive).

“The table was scrubbed” (patient, manner, passive).

“Victoria scrubs the table” (agent, manner, patient, transitive).

“Victoria cleans the table” (agent, result, patient, transitive).

“Victoria scrubs the table clean” (agent, manner, patient, result, transitive).

Different languages have different preferences for which construals are expressed. For so‐called verb‐framed languages (Talmy, 1985), for example, Romance languages, agent‐result‐patient constructions are the most typical. An example from Spanish is “La botella entra en la cueva” (The bottle enters the cave). On the other hand, so‐called satellite‐framed languages, for example, Germanic languages, agent‐action‐patient constructions are preferred. The event above is typically described in English as “The bottle is floating into the cave.” In the last example, the preposition “into” expresses the result vector. If one wants to add the action to the Spanish example, it becomes “La botella entra en la cueva flotando” (the bottle enters the cave floating).

Here, I have only given some simple examples of how the event model can be used to explain features of syntax. I believe that this approach can be expanded in many directions, and it is my plan to pursue this theme in the future. One example is an analysis of different types of Aktionsart (Gärdenfors, 2024).

## 5 Summarizing the semantic program

The basis of my semantic program is the theory of conceptual spaces. The components of the event model—agent, patient, force vector, results vector—can all be described in terms of conceptual spaces. As a summary, I outline how the structures can be used as foundations to answer a number of fundamental questions that seldom are treated by linguists.

1. How can children learn words so efficiently?

Bloom (2002) provides an excellent survey of research concerning how children learn the meaning of words. However, the enigma of how this learning can be achieved so quickly remains unanswered. In Sections 2.4 and 3, I have presented some mechanisms that can provide a solution to the enigma—at least a partial one. One mechanism is that a word is marked by syntactic cues that indicate which word class it belongs to. If it is an adjective, the child should look for a property in some domain; if it is a noun, there are a cluster of properties that hang together; if it is a manner verb, the child should find an appropriate action; if it is a result verb, the child should look for changes in some property; and so forth. The universal single‐domain hypothesis claims that for all word classes, except nouns, there is a region in some domain that represents the meaning of a word. Once the domain has been identified, the appropriate region can be approximately determined given only a few examples as shown in Section 2.4.

2. Why are words in languages divided into classes?

I propose that common word classes can be explained by how we mentally represent events. The different components of the event model are expressed by different word classes. Nouns refer to agents and patients. In order to identify unique referents, they are sometimes modified by adjectives. Force and result vectors are expressed by manner and result verbs, respectively. In cases where the result vector is a change of location (also metaphorically), the result vector can also be expressed by a prepositional phrase. Adverbs can be used to modify the force and result vectors. The use of pronouns follows principles of economy of expression since they make repetitions of noun phrases superfluous.

3. Why are sentences central units in language?

In all languages, sentences are fundamental units. Strangely enough, linguists have hardly considered why this is so. Philosophers, from Frege and on, answer that sentences express propositions, but propositions remain rather abstract entities that lack cognitive grounding. I have presented my answer in Section 4. In brief, I claim that sentences express events (states), and events are basic units of human causal thinking (Gärdenfors, 2024).

4. Why do sentences consist of noun phrases and verb phrases?

Again, it is taken for granted in linguistics that sentences have these two basic components, but there is no explanation for why this is so. As explained in Section 4.2, my answer to this question also derives from the structure of mental models of events: Agents and patients are described by noun phrases, and force vectors and result vectors are described by verb phrases.

## 6 Further applications of the theory of conceptual spaces

In this article, my focus has been to show how the theory of conceptual space can be used to generate a rich theory of semantics that is cognitively grounded. One benefit of the geometric and vectorial representations that are used in this program is that they are amenable to computational implementations and can therefore become useful in communication between humans and robots and other artificial intelligence systems (Gärdenfors, 2019). This is an application area that has great potential.

A second application area concerns *reasoning* with concepts. Despite significant theoretical and empirical progress in psychology, philosophy, and cognitive science, there has not been a unified framework for understanding how the structure of concepts influences how they are used in reasoning. Together with Matías Osta‐Vélez, I have embarked on a research program, where we argue that the theory of conceptual spaces is capable of filling this gap (Gärdenfors & Osta‐Vélez, 2023).

Our strategy is to demonstrate how various inference mechanisms that clearly rely on information about conceptual structures—including similarity, typicality, and diagnosticity‐based reasoning—can be modeled using principles derived from conceptual spaces. We first analyzed the role of *expectations* in inductive reasoning and their relation to the structure of concepts (Osta‐Veléz & Gärdenfors, 2022a). We have examined the relationship between using *generic* expressions in natural language and common‐sense reasoning as a second topic (Gärdenfors & Osta‐Vélez, 2024). We propose that the strength of a generic can be described by distances between properties and prototypes in conceptual spaces. Our third topic is *category‐based induction*. We demonstrate that the theory of conceptual spaces can serve as a comprehensive model for this type of reasoning (Osta‐Vélez & Gärdenfors, 2020). The final topic is *analogy* (Osta‐Vélez & Gärdenfors, 2022b). We present a taxonomy of analogical relations and show how to model them in terms of distances in conceptual spaces. We also discuss the implications of the model for reasoning with concepts in artificial systems.

A third application area concerns the structure and dynamics of scientific theories. Frank Zenker and I have interpreted laws of physics as geometrical rather than linguistic entities. Relying on conceptual spaces as a modeling tool, we show how to describe theory structures and how to evaluate their continuity (Gärdenfors & Zenker, 2013; Zenker & Gärdenfors, 2015, 2016). We focus on the conceptual framework an empirical theory presupposes, thus obtaining a geometrical representation of the structure of a theory. We stress the relevance of measurement procedures in separating conceptual from empirical structures. This lets our understanding of scientific laws come closer to scientific practice.

In conclusion, I have presented these examples of applications of conceptual spaces to show that the theory is productive and can be applied to a wide variety of areas involving concepts.

## Notes

1 Nosofsky (1992) presents an excellent survey of different mathematical models in this area.

2 For the distinction between integral and separable dimensions, see Garner (1974).

3 As described in Section 2.2, the brain implements such geometrical structures with the aid of grid cells in the entorhinal cortex.

4 See Paradis (2005) and Ravid (2006) for more detailed classifications of nouns.

5 Possible exceptions to this general rule are verbs that describe changes in ontology like “break” and verbs like “give” that describe intentional actions involving recipients. These are discussed in Gärdenfors (2014, section 10.3.2).

6 In Gärdenfors (2024), I show how to analyze events such as *remaining* in a state because the force vector is balanced by a counterforce that leads to no change.

7 More complex force vectors are represented in Croft's (2012) treatment of verbs.
